# Mediterranean Diet Adherence and Oxidative Stress in Autosomal Dominant Polycystic Kidney Disease: A Cross-Sectional Analysis of sNOX2-dp and Hydrogen Peroxide Concentration

**DOI:** 10.3390/antiox15010084

**Published:** 2026-01-08

**Authors:** Luca Salomone, Danilo Menichelli, Vittoria Cammisotto, Valentina Castellani, Pasquale Pignatelli, Francesca Tinti, Silvia Lai

**Affiliations:** 1 Department of Translational and Precision Medicine, Nephrology Unit, Sapienza University of Rome, 00185 Rome, Italy; 2 Department of General Surgery, Surgical Specialties and Organ Transplantation “Paride Stefanini”, Sapienza University of Rome, 00185 Rome, Italy; 3 Department of Clinical Internal, Anesthesiological and Cardiovascular Sciences, Sapienza University of Rome, 00185 Rome, Italyvalentina.castellani@uniroma1.it (V.C.);

**Keywords:** oxidative stress, Med-diet, autosomal dominant polycystic kidney disease, ADPKD, sNOX2-dp, H_2_O_2_, chronic kidney disease

## Abstract

Autosomal dominant polycystic kidney disease (ADPKD) is a genetic disorder marked by progressive kidney enlargement and cyst formation, often resulting in end-stage renal disease (ESRD). Oxidative stress (OxS) significantly contributes to renal damage in chronic kidney disease (CKD) and ADPKD. While the Mediterranean diet (Med-diet) is known for its antioxidative and anti-inflammatory effects, its impact on OxS in ADPKD remains unclear. This study aimed to assess the relationship between adherence to the Med-diet, OxS levels, and renal function in ADPKD patients. We enrolled 63 ADPKD patients aged 18–70 years with CKD stages G2–G4. Adherence to the Med-diet was evaluated using the PREDIMED questionnaire. OxS markers (NOX2-derived peptide [sNOX2-dp] and hydrogen peroxide [H_2_O_2_]) were measured via ELISA. Correlations between these markers, Med-diet adherence, serum creatinine, and estimated glomerular filtration rate (eGFR) were analyzed. Higher adherence to the Med-diet was associated with significantly lower OxS markers (sNOX2, *p* < 0.001; H_2_O_2_, *p* = 0.04). Reduced NOX2 and H_2_O_2_ levels correlated with lower creatinine and higher eGFR (NOX2, *p* < 0.001; H_2_O_2_, *p* < 0.001), suggesting an inverse relationship between OxS and renal function. In conclusion, adherence to the Mediterranean diet appears to be associated with lower levels of oxidative stress and may slow the progression of chronic kidney disease. These findings suggest that dietary interventions could mitigate disease progression by modulating OxS. Further studies are needed to confirm these results and explore the long-term effects of the Med-diet on disease progression.

## 1. Introduction

Autosomal dominant polycystic kidney disease (ADPKD) is the most common hereditary kidney disease leading to kidney failure [1]. Two genes are associated with ADPKD (PKD1 and PKD2), six with also autosomal dominant polycystic liver disease (ADPLD) (PRKCSH, SEC63, LRP5, ALG8, and SEC61B), and one associated with both (GANAB) [2]. The most frequent mutations occur in the PKD1 and PKD2 genes, encoding polycystin 1 and 2, respectively, which are integral membrane proteins located on the primary cilium of tubular cells. These proteins regulate calcium influx into the cell [3]. Mutations in PKD1 account for at least 80% of diagnoses, while PKD2 mutations are observed in 5% to 15% [2,4]. The prevalence of ADPKD is estimated to range from 1 in 400 to 1 in 1000 live births, affecting over 10 million people globally across all ethnic groups, making it a significant public health burden [1]. Renal manifestations of ADPKD include the gradual development of bilateral cysts and increased kidney size, which progressively lead to end-stage renal disease (ESRD) at an average age of 50, necessitating replacement therapy if untreated [5]. ADPKD also causes extrarenal manifestations such as hypertension, cerebral aneurysms, endothelial dysfunction, and an increased risk of cardiovascular disease (CVD), which remains a leading cause of morbidity and mortality in these patients. Oxidative stress (OxS) plays a key role in this process by promoting endothelial dysfunction, inflammation, and vascular remodeling. Elevated levels of OxS correlate with impaired renal function and increased cardiovascular risk, which appears to increase even in the early stages of the disease [6,7]. An overproduction of reactive oxygen species (ROS) and reactive nitrogen species (RNS) can lead to the oxidation of biological molecules such as lipids, proteins, and DNA, ultimately causing functional damage to multiple organs [8]. The kidney is a highly metabolic organ, making it particularly susceptible to OxS-induced damage. Several studies have demonstrated that OxS accelerates the progression of chronic kidney disease (CKD) and increases cardiovascular risk by depleting antioxidants and promoting excessive free radical production [9]. Recent evidence suggests that adherence to the Mediterranean diet (Med-diet) may help reduce ROS and overall OxS levels in various populations [10,11]. In particular, in patients with ESRD and an estimated glomerular filtration rate (eGFR) < 15 mL/min/1.73 m^2^, the Med-diet has shown potential benefits. Mekki et al. [12] demonstrated its role in reducing inflammation and peroxidation pathways, which may serve as a biological basis for lowering the risk of conditions such as hypertension, diabetes, cardiovascular diseases [13], and even cancer [9,14]. Additionally, Med-diet adherence has been associated with a slower annual decline in GFR in both CKD [15,16] and non-CKD populations [8,14].

There is limited evidence on the effects of the Mediterranean diet in patients with autosomal dominant polycystic kidney disease (ADPKD); however, some considerations can be drawn based on the available data in chronic kidney disease (CKD).

The Mediterranean diet is associated with a reduced risk of CKD progression and cardiovascular benefits in the general population and in patients with kidney disease, but no ADPKD-specific clinical outcome data are available [17,18]. A recent review confirms that the Mediterranean diet lowers the risk of incident CKD and may help preserve kidney function, although there is no evidence of a direct impact on mortality or on disease progression in ADPKD [19].

An observational study in ADPKD patients suggests that greater adherence to a healthy plant-based diet (which shares many features with the Mediterranean diet) is associated with better kidney function and a lower prevalence of advanced CKD [20]. However, the literature highlights that nutritional recommendations for ADPKD are largely based on general CKD principles [21,22]. In summary, the Mediterranean diet can be considered safe and potentially beneficial in ADPKD patients—particularly for preventing metabolic and cardiovascular complications—but no data are currently available on the impact of Med-diet adherence on OxS status and its influence on kidney disease progression in ADPKD patients. Our study was designed to assess OxS status in relation to Med-diet adherence in ADPKD. We focused on two circulating OxS biomarkers—serum soluble NOX2-derived peptide (sNOX2-dp) and hydrogen peroxide (H_2_O_2_)—as measures of systemic oxidative stress. These biomarkers were selected because sNOX2-dp reflects activation of NADPH oxidase 2 (a major ROS-generating enzyme), and H_2_O_2_ is a stable ROS indicating overall oxidative burden; both have been validated as OxS indicators in other populations (including CKD), although they have not been previously studied in ADPKD. Therefore, the aim of our study is to investigate OxS markers in relation to Med-diet adherence in ADPKD patients and to explore the connection between OxS and renal function.

### Aim of the Study

The primary endpoint of this study is to evaluate the relationship between OxS and adherence to the Med-diet in patients with ADPKD and CKD. OxS was estimated by the measurement of OxS biomarkers such as serum level of extracellular NOX2 fragment (sNOX2-dp) and hydrogen peroxide (H_2_O_2_).

The secondary endpoint was to evaluate the relationship between kidney function, expressed as eGFR and creatinine serum levels and oxidative stress biomarker serum levels (sNOX2-dp and H_2_O_2_).

## 2. Materials and Methods

### 2.1. Study Design and Subjects

We performed an observational, monocentric study on 63 consecutive ADPKD and CKD patients at the University Hospital “Policlinico Umberto I” of Rome, Sapienza University of Rome, Italy. Patients have been enrolled from June to November 2024. The study was approved by the local ethic committee of Sapienza University (No 7669 Prot. 0809/2024) on the 11 September 2024 and was conducted according to the 1975 Declaration of Helsinki. All patients signed informed written consent at study entry.

### 2.2. Inclusion Criteria

Patients aged 18–70 years with ADPKD (defined according to the Pei’s criteria [10]) and G2 to G4 stages of CKD (15 mL/min/1.73 m^2^ ≤ eGFR ≤ 90 mL/min/1.73 m^2^), calculated using the CKD-EPI equation according to Kidney Disease Improving Global Outcomes (KDIGO) guidelines, were recruited [11].

### 2.3. Exclusion Criteria

Patients with an age over 70 years, or CKD stage G5 or renal replacement therapy were excluded from this study. Furthermore, we excluded also patients with renal transplant recipients and patients with a clinical indication to follow a specific diet aimed at delaying CKD progression (e.g., low-protein or very-low-protein diets, use of ketoanalogues). Moreover, patients that refused to give consent and with missing data were also excluded.

### 2.4. Clinical and Laboratory Measurement

During the initial clinical examination, a comprehensive medical history was obtained, including comorbidities and cardiovascular and renal risk factors such as arterial hypertension, diabetes mellitus, heart failure, and metabolic syndrome. Anthropometric measurements (weight, height, and body mass index) were recorded, and routine blood tests were performed, including a complete blood count, creatinine, urea, uric acid, lipid profile, and serum electrolytes (sodium, potassium, calcium, phosphorus, and magnesium), as well as parathyroid hormone and vitamin D levels. Additionally, patients’ smoking habits and current pharmacological treatments were documented.

### 2.5. OxS Measurement

Between 8:00 a.m. and 9:00 a.m., after overnight fasting (12 h) and supine rest for at least 10 min, all the patients underwent blood samples. Blood samples obtained from patients were collected into tubes (Vacutainer Systems, Belliver Industrial Estate, Plymouth, UK) with or without anticoagulant (3.8% sodium citrate) and were centrifuged at 300× *g* for 10 min at room temperature to obtain supernatant. For serum isolation blood was drawn and allowed to clot at room temperature for 1 h. Serum and plasma samples were immediately stored at −80 °C until analysis.

To analyze OxS, blood samples were taken from ADPKD patients in fasting conditions.

NOX2 is a isoform of the NADPH oxidase complex, a family of enzymes producing ROS [23]. To evaluate NOX2 activity, we measured serum levels of soluble NOX2-derived peptide (sNOX2-dp), a marker of NOX2 activation, by ELISA method as previously described [24]. Values were expressed as pg/mL; intra and inter-assay coefficients of variation were 5.2% and 6%, respectively.

Hydrogen peroxide (H_2_O_2_) in serum samples was assessed by a Colorimetric Detection Kit (Arbor Assays, Ann Arbor, MI, USA) as previously described [25]. Values were expressed as μM. Intra-assay and inter-assay coefficients of variation were 2.1% and 3.7%, respectively.

### 2.6. Med-Diet Definition and Classification

The Mediterranean diet emphasizes foods commonly consumed in Mediterranean countries, favoring cereals, fruits, vegetables, seeds, and olive oil while limiting red meats and animal fats. It also includes moderate consumption of fish, poultry, legumes, eggs, dairy, red wine, and sweets. At enrolment, patients completed the PREDIMED questionnaire to assess adherence to the Mediterranean diet. The PREDIMED questionnaire is a simple and practical tool designed to assess how closely a person follows the eating habits typical of the Mediterranean diet [26,27]. The questionnaire evaluates the limitation of less healthy foods such as red meat, processed meats, sweets, and sugary drinks. Each response is worth 1 point if the dietary habit aligns with Mediterranean diet criteria, or 0 points if it does not. The total score ranges from 0 to 14, allowing classification of adherence to the diet as follows:

• 0–5 indicates low adherence,
• 6–9 moderate adherence,
• 10–14 high adherence.

Thanks to its simplicity, the PREDIMED questionnaire is highly useful in both clinical practice and research. It enables the rapid identification of dietary habits that could be improved and provides a foundation for correlating adherence to the Mediterranean diet with various health indicators, such as oxidative stress regulation, cardiovascular disease prevention, and the management of chronic conditions like kidney disease [28,29,30].

### 2.7. Statistical Analysis

Categorical variables were reported as numbers and percentages which were compared with Pearson’s χ^2^ test. Mean and standard deviation (SD) or median and interquartile range (IQR) were used for continuous variables, which were compared by Student’s t-test or the Mann–Whitney U test, respectively. Normal distribution of variables was checked by the Kolmogorov–Smirnov test. When multiple comparisons were performed, Bonferroni’s adjustment of α error was applied. We used Student’s unpaired t test and Pearson product–moment correlation analysis to evaluate normally distributed continuous variables and an appropriate nonparametric test (Mann–Whitney U test and Spearman rank correlation test) for the other variables. Group comparisons were performed using Fisher’s F-test (ANOVA) or Kruskal–Wallis test when needed. Clinical and laboratory characteristics were described according to total Med-Diet score tertiles.

All tests were 2-tailed and only *p*-values < 0.05 were considered statistically significant. The analyses were performed using SPSS 25.0 software (IBM, Armonk, NY, US) and GraphPad software, version 10.6.1.

### 2.8. Sample Size Calculation

According to previous study performed by Pastori et al. [31] and estimating a reduction of sNOX2-dp at least 15% with a power of 80% and α error = 0.05, the sample size was 56 patients.

## 3. Results

### 3.1. Baseline Characteristics

We enrolled 63 consecutive patients with ADPKD. Anthropometric data and serum metabolite levels are summarized in Table 1. There were no significant differences among tertiles or between the first and third tertiles about age, sex, comorbidities, and laboratory findings (Table 1), except for mean eGFR, mean serum creatinine, sNOX2-dp, and H_2_O_2_ (Table 1).

### 3.2. Med-Diet and OxS

**Figure 1:**
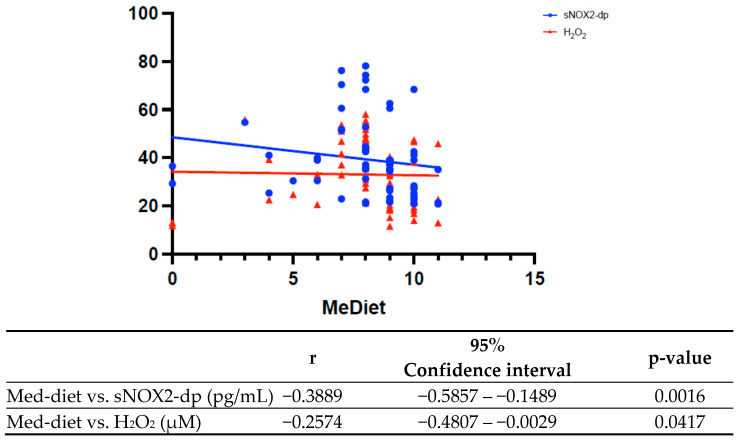
Spearman correlation between Mediterranean diet and oxidative stress biomarkers.

At Spearman analysis, an inverse correlation was found between adherence to the Med-diet and serum levels of sNOX2-dp (Figure 1, r = −0.3889, 95% confidence interval [95%CI]: −0.5857–−0.1489, *p* = 0.0016) and between adherence to the Med-diet and H_2_O_2_ serum levels (Figure 1, r = −0.2574, 95%CI: −0.4807–−0.002859, *p* = 0.0417).

### 3.3. OxS and Kidney Disfunction

**Figure 2:**
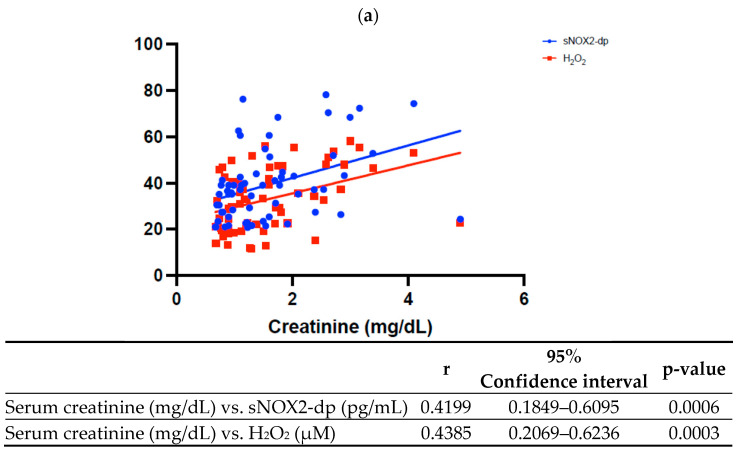

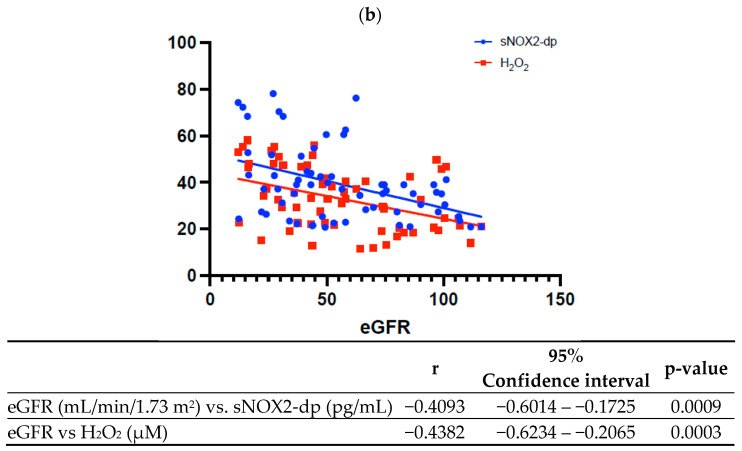
Spearman correlation between oxidative stress biomarkers and serum creatinine (**a**) and estimated glomerular filtration rate (eGFR) (**b**).

The Spearman analysis showed a direct correlation between serum creatinine and sNOX2-dp (Figure 2a, r = 0.4199, 95%CI: 0.1849–0.6095, *p* = 0.0006) and between serum creatinine and H_2_O_2_ (Figure 2a, r = 0.4385, 95%CI: 0.2069–0.6236, *p* = 0.0003).

Higher OxS levels were associated with lower eGFR, confirmed by the inverse correlation observed at Spearman analysis: indeed, eGFR was inversely correlated with both serum levels of sNOX2-dp (Figure 2b, r = −0.4093, 95%CI: −0.6014–−0.1725, *p*= 0.0009) and H_2_O_2_ (Figure 2b, r = −0.4382, 95%CI: −0.6234–−0.2065, *p*= 0.0003).

## 4. Discussion

Oxidative stress is widely recognized as a key contributor to kidney damage, as ROS can drive renal disease through multiple mechanisms. ROS directly injure glomerular cells, triggering inflammation and fibrosis, while also activating signaling pathways and transcription factors that further exacerbate these processes. Renal OxS is closely linked to diabetes, hypertension, and metabolic syndrome, contributing to glomerular hypertension, endothelial and epithelial cell damage, and albuminuria [32,33]. The tubulointerstitial region is particularly vulnerable to OxS-related injury. Tubulointerstitial damage, often following acute kidney injury (AKI), significantly increases the risk of CKD progression. Within this region, oxidative damage to peritubular capillary endothelial cells can lead to tubular cell injury, partly through an imbalance in the ROS-to-NO ratio. This, in turn, activates inflammatory and fibrotic signaling pathways, including NF-κB, inflammasomes, and Wnt–β-catenin [34]. Given these mechanisms, the hypothesis that OxS contributes to reduced kidney function in ADPKD is highly plausible. However, to date, no studies have directly compared OxS levels in ADPKD with other forms of CKD. It is reasonable to speculate that ADPKD patients may experience higher OxS levels due to the disease’s intrinsic pathophysiology. An interesting perspective on ADPKD disease progression comes from the KETO-ADPKD study, which evaluated the impact of a ketogenic diet on slowing kidney disease progression. Surprisingly, in patients adhering to the ketogenic diet, not only was the decline in eGFR slowed, but an improvement in kidney function over time was also observed [35]. These findings indicate that dietary patterns may be associated with trajectories of kidney function in ADPKD; however, evidence varies by intervention and setting, and generalizability to other dietary patterns is uncertain. However, no direct evidence currently exists regarding the potential benefits of the Med-diet in this context. In our study, we observed reduced values of serum creatinine associated with lower levels of NOX2 and H_2_O_2_, which correlated with higher adherence to the Med-diet. These cross-sectional findings indicate that higher adherence to the Med-diet was associated with a more favourable oxidative stress profile and contemporaneous kidney function indices; whether these associations translate into differences in CKD progression requires longitudinal and interventional studies. Our data indicate that ADPKD patients with greater adherence to the Med-diet exhibit significantly lower OxS levels, which, in turn, correlate with better kidney function. Specifically, reduced serum NOX2dp levels are associated with higher GFR and lower serum creatinine, indicating that lower OxS marker levels were observed among participants with higher eGFR and lower serum creatinine at the time of testing. Moreover, Bacharaki et al. showed that greater adherence to the Med-diet is linked to reduced left ventricular hypertrophy, a major cardiovascular risk factor. By preserving normal cardiac geometry, the Med-diet may offer protection against cardiac dysfunction in CKD patients [36]. In addition, Kwon et al. showed that the Med-diet is safe even in patients with advanced CKD, as it does not adversely affect serum and urine potassium levels while helping to maintain kidney function [37]. Furthermore, a meta-analysis by Hansrivijit et al. revealed that adherence to the Med-diet was associated with a 10% lower risk of developing CKD. However, data on patients with pre-existing CKD or those undergoing dialysis remain insufficient [38]. In recent years, OxS has emerged as a crucial factor in the pathophysiology of ADPKD, contributing to both cystogenesis and disease progression; in fact, in ADPKD, several mechanisms contribute to increased oxidative damage, including mitochondrial dysfunction that lead to excessive ROS production [33]. Mitochondrial dysfunction plays a critical role in ADPKD by disrupting cellular energy homeostasis, thereby exacerbating oxidative damage and inflammation [39]. Other studies have shown increased NOX activity in ADPKD, promoting oxidative damage, fibrosis, and cyst expansion. Furthermore, increased NOX enzyme activity has been observed in ADPKD, promoting OxS, fibrosis, and cyst expansion. Targeting OxS pathways may offer therapeutic benefits, as evidenced by studies showing that NRF2 activation can mitigate oxidative damage and slow cystogenesis [40]. Additionally, OxS and inflammation are closely interconnected in ADPKD. Persistent oxidative damage activates inflammatory pathways, leading to the release of cytokines such as TNF-α, IL-6, and MCP-1, which further contribute to renal injury and fibrosis [41]. Also, ADPKD patients often exhibit reduced levels of endogenous antioxidants, such as superoxide dismutase (SOD) and glutathione, making renal cells more susceptible to oxidative damage [42]. Notably, OxS and endothelial dysfunction are evident even in the early stages of ADPKD, even when renal function is still preserved [42]. Lifestyle interventions such as regular physical activity and calorie restriction have been associated with improved mitochondrial function and reduced OxS in kidney diseases. Additionally, the Med-diet, rich in antioxidants and polyphenols, has been shown to counteract oxidative stress and inflammation, potentially benefiting ADPKD patients [33]. Oxidative stress plays a central role in the pathogenesis and progression of ADPKD, contributing to cyst growth, inflammation, and renal fibrosis. Understanding the mechanisms linking OxS to ADPKD may open new avenues for therapeutic interventions, the potential clinical relevance of dietary pattern assessment in ADPKD, while underscoring the need for prospective and interventional studies.

### Limitations

This study has several limitations that should be considered when interpreting the results. Given its observational design, the findings should be regarded as associative, and no causal inferences can be drawn regarding the relationship between Med-diet adherence, oxidative stress biomarkers, and kidney function.

The sample size was relatively small (n = 63), which may have limited statistical power, increase the risk of type II error, and reduced the precision and generalizability of the estimates.

The cross-sectional nature of the analysis precludes assessment of temporal relationships and does not allow evaluation of long-term effects or clinical outcomes, such as changes in eGFR over time or progression to advanced CKD stages. Fourth, because participants were recruited from a single center, selection bias cannot be excluded and external validity may be limited, as the cohort may not be fully representative of the broader ADPKD population in different clinical settings. Furthermore, the known susceptibility of H_2_O_2_ colorimetric assays to analytical noise, or the potential influence of platelet or endothelial activation on sNOX2-dp levels may potentially affect the results.

In addition, the observational design of our study, unlike clinical trial, we could not evaluate biomarkers of adherence. However, this is common and coherent with previous observational studies [13,43,44], in which adherence to the MedDiet was recorded and evaluated only according to structured interview performed by medical team to patients.

Our study may be considered as exploratory and a hypothesis-generator with preliminary findings: indeed, the low sample size did not allow to adjust for potential confounders due to high overadjustment risk of the analysis.

Finally, although we considered relevant clinical variables, residual confounding remains possible. Potentially important factors were not systematically evaluated, including physical activity, psychosocial stress, smoking intensity, alcohol intake blood pressure control, and sleep quality, which could influence both dietary patterns and oxidative stress or kidney function indices.

## 5. Conclusions

Our findings show that, in this cross-sectional cohort of patients with ADPKD, higher adherence to the Mediterranean diet was associated with a more favorable oxidative stress profile, as reflected by lower circulating levels of NOX2dp and H_2_O_2_. Moreover, these oxidative stress biomarkers were correlated with contemporaneous measures of kidney function, including lower serum creatinine and higher eGFR, indicating that OxS status and renal function are linked at the time of assessment. These observations are biologically plausible in consideration of accumulating evidence implicating oxidative stress, mitochondrial dysfunction, NOX activation, and inflammatory signaling pathways ADPKD-related renal injury, cystogenesis, and fibrotic remodeling, as well as in the broader context of CKD. Importantly, however, the present study design does not allow inference on causality or directionality; therefore, the associations observed here cannot establish that Med-diet adherence reduces oxidative stress or that lower oxidative stress mediates better kidney function. Reverse causation and residual confounding (including lifestyle factors, comorbidities, and treatment differences) remain plausible explanations.

Taken together, our results support the relevance of Mediterranean dietary patterns as correlates of oxidative stress and renal function in ADPKD and provide hypothesis-generating evidence that diet-related exposures may be meaningfully linked to pathophysiological pathways of interest in this disease. In parallel, external evidence in non-ADPKD populations suggests that Mediterranean diet adherence is associated with favorable cardio-renal phenotypes, including lower left ventricular hypertrophy and a reduced risk of incident CKD, and appears feasible and safe even in advanced CKD, strengthening the rationale for further investigation in ADPKD. Future work should prioritize adequately powered prospective cohorts to clarify temporal relationships and to reduce the risk of reverse causation, and randomized dietary interventions to test whether Mediterranean diet adherence can modify oxidative stress biomarkers and translate into clinically relevant renal outcomes (e.g., eGFR slope, albuminuria trajectories, and time-to-CKD progression endpoints). Standardized assessment of diet adherence, repeated biomarker measurements, and comprehensive adjustment for confounders will be essential to determine whether the observed associations reflect causal pathways and to define the potential role of dietary strategies within the long-term management of ADPKD.

## Figures and Tables

**Table 1:** Baseline characteristics of population according to Med-diet adherence.

	Total Cohort (n: 63)	1st Tertile	2nd Tertile	3rd Tertile	*p*-Value Overall	*p*-Value 1st vs. 3rd
Age (years)	50.0 ± 11.8	47.1 ± 12.8	51.5 ± 11.1	49.8 ± 12.5	0.509	0.560
Female sex (%)	66.7	53.3	65.6	81.3	0.253	0.097
Hypertension (%)	85.2	64.3	96.8	81.3	0.015	0.295
Smoking habits (%)	13.1	14.3	12.9	12.5	0.988	0.886
BMI	24.4 ± 4.5	25.4 ± 4.1	23.5 ± 4.2	25.6 ± 5.3	0.222	0.892
Anemia (%)	17.7	6.7	25.8	12.5	0.230	0.583
Mean Med-diet score	8.1 ± 2.2	5.1 ± 2.4	8.5 ± 0.5	10.2 ± 0.4	<0.001	<0.001
**Therapy**						
ACE-I/ARBs (%)	78.3	64.3	83.3	81.3	0.341	0.295
Beta blockers (%)	15.3	7.1	20.7	12.5	0.480	0.626
CCB (%)	45.8	38.5	51.6	40.0	0.635	0.934
Diuretics (%)	9.8	14.3	9.7	6.3	0.761	0.464
Tolvaptan/Octreotide (%)	22.2	13.3	34.4	6.3	0.056	0.505
**Laboratory findings**						
Haemoglobin (g/dL)	13.0 ± 1.5	13.5 ± 1.7	12.6 ± 1.4	13.3 ± 1.4	0.091	0.735
Platelets (×10^3^/µL)	233.5 ± 102.6	209 ± 63.5	231.2 ± 68.8	256.3 ± 167.5	0.508	0.384
Creatinine (mg/dL)	1.4 ± 0.6	1.2 ± 0.5	1.6 ± 0.6	1.1 ± 0.5	0.013	0.389
eGFR(mL/min/1.73 m^2^)	59.7 ± 26.8	65.3 ± 23.5	49.5 ± 22.4	75.6 ± 29.2	0.004	0.339
H_2_O_2_ (µM)	33.0 ± 13.3	34.5 ± 14.0	34.6 ± 13.3	28.5 ± 12.5	0.295	0.222
NOX2-dp (pg/mL)	39.3 ± 15.8	44.0 ± 16.3	41.4 ± 15.8	30.6 ± 12.5	0.032	0.015

ACE-I/ARBs: Angiotensin converting enzyme inhibitors/Angiotensin receptor blockers, BMI: body mass index, CCB: calcium channel blockers, eGFR: estimated glomerular filtration rate, H_2_O_2_: hydrogen peroxide, NOX2-dp: soluble NOX2-derived peptide.

## Data Availability

Data available on request due to privacy restrictions. The data presented in this study are available on request from the corresponding author.
